# Identification of a *Taenia* Tapeworm Carrier — Los Angeles County, 2014

**Published:** 2015-01-30

**Authors:** Curtis Croker, Jan Soriano, Rachel Civen, Robert A Larsen, Benjamin Schwartz

**Affiliations:** 1 Acute Communicable Disease Control Program, Department of Public Health, Los Angeles County, California; 2 LAC/USC Medical Center, Los Angeles, California

Carriers of the pork tapeworm, *Taenia solium*, are the sole source of cysticercosis, a parasitic tissue infection (1). When tapeworm eggs excreted by the carrier are ingested, tapeworm larvae can form cysts. When cysts form in the brain, the condition is called neurocysticercosis and can be especially severe. In Los Angeles County an average of 136 county residents are hospitalized with neurocysticercosis each year (2). The prevalence of *Taenia solium* carriage is largely unknown because carriage is asymptomatic, making detection difficult. The identification and treatment of tapeworm carriers is an important public health measure that can prevent additional neurocysticercosis cases (1).

On June 6, 2012, a woman aged 33 years in Los Angeles County who had emigrated from El Salvador in 2004 was diagnosed with hydrocephalus caused by a ventricular cystic lesion identified by magnetic resonance imaging. A ventriculo-peritoneal shunt was required. Neurocysticercosis was included in the differential diagnosis in 2012, but was not confirmed until July 21, 2014, when the patient had a positive serology for cysticercosis after testing by a CDC reference diagnostic laboratory.

A public health investigation identified seven persons who shared the woman’s household and who were screened for tapeworms. Each household member submitted three stool specimens for examination. A public health nursing practice model, based on nationally recognized components and using a population-based, team approach, was used to ensure compliance with stool specimen collection (3).

One household member, a woman aged 37 years, was identified as a *Taenia* tapeworm carrier. *Taenia* eggs were identified by light microscopy in one of her three stool specimens at the county public health laboratory. *Taenia* eggs of different species are morphologically indistinguishable. The carrier was in good health and reported no symptoms. She worked as a cashier at a bakery, but did not handle food. She reported no foreign travel since emigrating from Guatemala in 2005. The carrier was evaluated by an infectious disease physician and treated with a single, 600-mg dose of praziquantel. She was instructed to collect any worm segments from her stool within 3 days after treatment so that the *Taenia* species could be identified; however, no tapeworm segments were identified. One month after treatment, the carrier was again screened for *Taenia*. No evidence of *Taenia* was found in any of the three stool specimens examined, and the carrier was considered cleared of infection.

Identification of *Taenia* tapeworm carriers by screening household members (including housekeepers) of patients with neurocysticercosis in the United States has been reported (4–7). Clinicians need to consider neurocysticercosis in patients with cystic cerebral lesions and report neurocysticercosis cases to their local health department so that they can investigate the cases and screen all household members for tapeworms.

## References

[1] CDC (2014). Parasites—cysticercosis.

[2] Croker C, Redelings M, Reporter R (2012). The impact of neurocysticercosis in California: a review of hospitalized cases. PLoS Negl Trop Dis.

[3] County of Los Angeles Department of Public Health Nursing Administration Public health nursing model.

[4] Schantz PM, Moore AC, Muñoz JL (1992). Neurocysticercosis in an orthodox Jewish community in New York City. N Engl J Med.

[5] Asnis D, Kazakov J, Toronjadze T (2009). Case report: neurocysticercosis in the infant of a pregnant mother with a tapeworm. Am J Trop Med Hyg.

[6] O’Neal S, Noh J, Wilkins P (2011). *Taenia solium* tapeworm infection, Oregon, 2006–2009. Emerg Infect Dis.

[7] Sorvillo FJ, Waterman SH, Richards FO, Schantz PM (1992). Cysticercosis surveillance: locally acquired and travel-related infections and detection of intestinal tapeworm carriers in Los Angeles County. Am J Trop Med Hyg.

